# Adjunct Therapy With Glycyrrhiza Glabra Rapidly Improves Outcome in Depression—A Pilot Study to Support 11-Beta-Hydroxysteroid Dehydrogenase Type 2 Inhibition as a New Target

**DOI:** 10.3389/fpsyt.2020.605949

**Published:** 2020-12-10

**Authors:** Harald Murck, Lisa Lehr, Johannes Hahn, Matthias C. Braunisch, Daniela Jezova, Maxim Zavorotnyy

**Affiliations:** ^1^Department of Psychiatry and Psychotherapy, Philipps-University Marburg, Marburg, Germany; ^2^Murck-Neuroscience, Westfield, NJ, United States; ^3^Department of Nephrology, Klinikum Rechts der Isar, School of Medicine, Technical University Munich, Munich, Germany; ^4^Institute of Experimental Endocrinology, Biomedical Research Center, Slovak Academy of Sciences, Bratislava, Slovakia; ^5^Department of Psychiatry and Psychotherapy, Psychiatric Services Aargau, Academic Hospital of the University of Zurich, Brugg, Switzerland; ^6^Marburg Center for Mind, Brain and Behavior—MCMBB, University of Marburg, Marburg, Germany

**Keywords:** major depression, aldosterone, mineralocorticoid receptor, inflammation, depression subtypes, toll like receptor 4 (TLR4), cortisol

## Abstract

Mineralocorticoid-receptor (MR) dysfunction as expressed by low systolic blood pressure and a high salivary aldosterone/cortisol ratio predicts less favorable antidepressant treatment outcome. Inhibition of peripheral 11-beta-hydroxysteroid-dehydrogenase type 2 (11betaHSD2) reverses these markers. We therefore tested the hypothesis that the 11betaHSD2 inhibitor glycyrrhizin affects treatment outcome via this mechanism. We administered Glycyrrhiza glabra (GG) extract containing 7–8 % of glycyrrhizin at a dose of 2 × 700 mg daily adjunct to standard antidepressants in hospitalized patients with major depression. These subjects were compared in an open-label fashion with patients, who did not receive GG (treatment as usual, TAU). Assessments were done at baseline and approximately 2 weeks after. Twelve subjects were treated with GG and compared to 55 subjects with TAU. At week 2, the Hamilton Depression Rating Scale (HAMD-21) change from baseline as well as the CGI-S change showed a significant time × treatment interaction (*p* < 0.03), indicating a possible therapeutic benefit of GG. Clinical benefit seems to be more pronounced in subjects with lower systolic blood pressure and significantly correlated with reduced sleep duration in the GG group. Our preliminary data show that treatment with the 11betaHSD2 inhibitor glycyrrhizin may possess a beneficial effect on antidepressant response, which may be specific to a defined depression subtype.

## Introduction

Major depressive disorder (MDD) is a very common mental condition with a lifetime prevalence of around 15%. According to the largest prospective trial (Star^*^D), approximately only one third of all subjects achieved remission within 6 weeks of standard treatment (1). In previous reports, neuroendocrine changes in major depressive disorder (MDD) have been widely described, which mainly focused on the stress hormone cortisol. Besides cortisol, the involvement of aldosterone in the stress response became increasingly evident in recent years. It was, however, already stated by the founder of the stress concept Hans Selye (2). More recently, aldosterone has been recognized as a potential biomarker of the depressive state as supported by clinical (3–5) and preclinical data (6–10). Of importance is that markers of increased aldosterone action via central mineralocorticoid receptors appear to be associated with therapy resistance to standard antidepressants (11, 12). Proposed mechanisms include an interaction with aldosterone specific brain areas, including the nucleus of the solitary tract (see below) (13, 14) and of aldosterone-induced inflammation (9, 15, 16). The increased aldosterone concentration in treatment refractory patients is associated with a *reduced* systolic blood pressure, which points to a reduced peripheral sensitivity of the mineralocorticoid receptor (MR) to aldosterone (11). Thus, reduced peripheral MR sensitivity may be related to treatment refractoriness in MDD, as independently described (17, 18).

A recent study demonstrated in a placebo-controlled randomized trial the added benefit of glycyrrhizin as an adjunct treatment to SSRI (19). Interestingly, glycyrrhizin targets exactly the biological pattern, which we identified in our bottom up approach to define therapy refractory depression (TRD): it reduces circulating aldosterone (and vasopressin) levels (20–23) and also increases blood pressure (24) in a dose-dependent manner. A demonstrated molecular mechanism is the inhibition of the enzyme 11-beta hydroxysteroid-dehydrogenase type 2 (11betaHSD2) in the periphery (24, 25). The inhibition of 11betaHSD2 allows cortisol to reach the intracellular MR (26), which is otherwise metabolized by the enzyme. This is crucial for aldosterone's specificity, as aldosterone and cortisol have a similar affinity at the MR, but cortisol has an ~500-fold higher plasma concentration. An inhibition of this enzyme therefore allows cortisol to act as a mineralocorticoid (27). A feedback mechanism, via an MR-mediated inhibition of renin release, leads consequentially to a reduced aldosterone secretion in combination with an increased blood pressure. Furthermore, glycyrrhizin has anti-inflammatory effects not only by reducing plasma aldosterone but also by direct antagonistic effects at the toll-like receptor 4 (TLR4), which is a crucial trigger for innate immunity (28–30). The fact that polymorphisms of the 11betaHSD2 gene exist and are involved in blood pressure regulation (27) provides a potential basis for interindividual differences, which might become the basis for a future personalized treatment approach.

With regard to the central action of aldosterone, it should be noted that aldosterone binds only to specific binding locations within the brain, despite the wide distribution of the MR. Most brain MRs, for example the classical intracellular MRs in the hippocampus, are not readily accessible to aldosterone. As stated above, this is due to the fact that cortisol and aldosterone have a similar affinity to the MR, but cortisol is present in an ~500-fold higher concentration in the blood stream (31). MR-selective tissue in the brain requires the intracellular presence of 11betaHSD2, similarly to the situation in the periphery (31). This is not the case in the hippocampus. Instead, the nucleus tractus solitarii (NTS), which expresses both the MR and the 11betaHSD2, is the primary anatomical target of aldosterone in the brain (32).

Taken together, there is a growing body of evidence demonstrating an association of peripheral MR dysfunction, increased aldosterone levels, and TRD. Also, an inhibition of peripheral 11betaHSD2 appears to activate peripheral MR and reduce aldosterone release. Therefore, we hypothesize that add-on glycyrrhizin, which is provided in the form of a glycyrrhizin-containing extract from Glycyrrhiza glabra (GG), inhibits peripheral 11betaHSD2 and as a consequence may expand the effectiveness of antidepressant drugs. Our current study compared a group of patients, who received GG adjunct to standard antidepressant treatment with a group, which only received treatment as usual (TAU). We were further interested in potential predictors and moderators of the response to glycyrrhizin, based on earlier identified biomarkers and in particular its action to increase peripheral MR activity, to reduce aldosterone secretion and therefore central MR activation.

## Methods

The studied patients were hospitalized at the Department for Psychiatry and Psychotherapy of the Philipps University Marburg. Subjects were recruited for an ongoing observational study [renin-angiotensin aldosterone system (RAAS) study], in which patients were treated with standard antidepressant treatment only, and a second study to examine the effects of GG on biomarkers and clinical characteristics of patients, who were treated with standard antidepressants and in addition received twice daily GG (GG study). Both studies were approved by the ethics committee of the Philipps-University Marburg. Participating patients, who met inclusion/exclusion criteria, signed an informed consent.

### Study Conduct

Before a new treatment regime was started (in one of the GG subjects, this consisted of a non-pharmacological treatment), a baseline assessment took place. Two days after the baseline examination, the open-label treatment with GG was started at a dose of 2 × 2 capsules of a standardized GG extract in the form of capsules from Glycyrrhiza glabra (GG) in the GG group. The extract contained 7–8 % of glycyrrhizin (Gall Pharma, Austria) and was administered at a dose of 2 × 700 mg daily adjunct to standard treatment to hospitalized patients with major depression. In addition, 2 × 150 mg magnesium in the form of magnesium citrate was administered in order to overcome potential long-term tolerability issues, which could occur due to the MR-mediated increased magnesium depletion. Capsules were taken in the morning and evening. Clinical and neurophysiological parameters were determined at baseline and 2 weeks after baseline. The changes from baseline in clinical and biomarker parameters were compared to the group receiving TAU.

### Patient Population

For inclusion, patients had to meet ICD-10 diagnostic criteria for major unipolar depressive disorder. Patients with psychosis or with severe personality disorders were excluded. For further information, the criteria are described more precisely in previous works (11). Demographic and medication use data are presented in Table 1. Despite the difference in the number of subjects, most of the patient characteristics are fairly balanced. Two notable exceptions are the numerically higher number of depressive episodes and lower frequency of the use of atypical neuroleptics. These differences are not expected to favorably bias the GG treatment group.

**Table 1:** Demographic and medication characteristics.

	**GG**			**TAU**		
	***N***	**Mean**	**Std**.	***N***	**Mean**	**Std**.
			**deviation**			**deviation**
Age (years)	12	37.3	10.9	55	44.6	15.7
Age of onset (years)	12	27.3	10.0	54	36.4	16.1
Number of episodes	12	7.4	8.1	51	3.4	4.0
Duration current episode (weeks)	12	25.1	16.9	51	23.5	39.4
BMI kg/m^2^	12	29.9	7.7	51	27.8	5.5
		**Number/(%)**			**Number/(%)**	
Gender m/f		7/5			23/32	
SSRI/SNRI	12	9 (75%)		55	44 (80.0%)	
Tricyclic	12	1 (8.3%)		55	4 (7.2%)	
Mirtazapin	12	0		55	12 (21.8%)	
MAO inhibitors	12	1 (8.3%)		55	3 (5.4%)	
Lithium	12	1 (8.3%)		55	8 (14.5%)	
Atypical neuroleptics	12	4 (25.0%)		55	24 (43.6%)	
Low dose typical neuroleptics	12	3 (25.0%)		55	16 (29.0%)	
None	12	1 (8.3%)		55	0	

### Collected Parameters

Three trained raters conducted all clinician ratings to reduce interrater variability. All ratings for a given subject were performed by the same rater. Clinician rating consisted of the Clinical Global Impression Scale for severity (CGI-S) (33) and Hamilton Depression Rating Scale with 21 items (HDRS-21) (34). In addition, scores for item 1 of the HAMD-21 (depressiveness) is reported.

Saliva for the measurement of cortisol and aldosterone was collected immediately after awakening. Patients had to consecutively chew on two Salivettes® Cortisol samplers (Sartstedt, Nümbrecht, Germany) in order to gain enough saliva. Samples were frozen at−20°C up to two weeks and then at−80°C until shipment by mail to the Institute of Experimental Endocrinology (Slovak Academy of Sciences, Bratislava, Slovakia) for analysis. Saliva cortisol concentrations were analyzed using a commercially available enzyme-linked immunosorbent assay (IBL International, Hamburg, Germany). Salivary aldosterone concentrations were measured by a modified methodology (35) using a commercial radioimmunoassay kit (Immunotech, Prague, Czech Republic). To get usable results, saliva was concentrated three times.

- Blood pressure was measured using an automatic blood pressure monitor (Omron M5 Professional™, Omron Medizintechnik, Mannheim, Germany) mounted on the patient's upper right arm.
- Heart rate variability was determined as respiratory sinus arrhythmia (RSA) two times with a device and corresponding app (iThlete®, HRV Fit Ltd, Hampshire, United Kingdom) linked to a smartphone (iPhone), as described in detail before (11). The measurement took place 5–10 min after awakening. Heart rate variability was measured twice for a 55-s test interval each. Patients followed the breathing cadence of 7.5 breaths per minute displayed by a lung animation on the smartphone screen. Calculations performed within the iThlete app are based on the time domain HRV index, which is the root mean square of successive R–R intervals (RMSSD). The mean of two sequential measurements is reported here.
- Sleep monitoring was carried out by a simplified polysomnographic system (Zeo™, Inc., Newton, MA, USA) that used only three proprietary sensors in a headband that transferred sleeping data wirelessly to a bedside monitor. The electrodes of the headband were placed at the forehead below Fp1, Fpz, and Fp2. Scoring took place in 30-s epochs each night using four sleep stages (wake, light sleep, deep sleep, and rapid eye movement sleep). The advantage of this monitoring system is that assessments are carried out in the usual sleeping environment, thus avoiding the common problems of sleep laboratory settings. Validity, as determined by correlation with an expert system, has been demonstrated for SWS and sleep duration.
- Blood draws for plasma electrolytes, including sodium, potassium, and magnesium, were conducted before noon. Plasma Na^+^ and K^+^ levels were measured using specific ionic selective electrodes. Mg^2+^ was measured photometrically at 520 nm after complexion with calmagite (3-hydroxy-4-[(2-hydroxy-5-methylphenyl)azo]-1-naphthalenesulfonic acid).

### Procedures at Assessment Days

The examinations were performed in the following sequence: In the evening at approximately 7 p.m., HDRS-21 and CGI were performed. This was followed by the adjustment of a headband which contained electrodes for a sleep EEG device. Immediately after awakening, saliva for the analysis of aldosterone and cortisol was collected. The sampling occurred before patients got out of bed. Immediately after that, the HRV assessment and a salt taste test were performed. The morning assessments were concluded by the determination of blood pressure and the self-rating scales BDI and QIDS (not reported here). Blood samples for the determination of electrolytes were taken on this day before noon, after at least 10 min of bedrest.

### Statistical Analysis

As a proof-of-concept analysis, we compared the change of the HAMD-21 score from baseline to week 2 in subjects, who took an adjunct treatment with GG to the TAU group. We used a repeated-measurement general linear model, using SPSS, version 25, with treatment, gender, and rater as factors. The same approach was used for the exploratory analysis of the biochemical, endocrine, and physiological parameters.

In order to characterize the potential relationship (1) between baseline parameters and clinical change and further (2) the change in biomarker parameters and clinical change, we used correlational analysis, utilizing Spearman correlation coefficients. For this analysis, the relative change of the HAMD-21 from baseline (R-HAMD-21) was expressed as a ratio of the HAMD-21 at week 2 divided to the baseline HAMD-21. For example, a 20% increase would lead to a factor of 1.2, a 20 % reduction to a factor of 0.8. This number was then correlated with the baseline parameters. A similar approach for the change of biomarkers was used. The *p*-values of the statistical tests are provided on the basis of a two-sided assessment. A *p* ≤ 0.05 is regarded as statistically significant.

### Power Calculation

A statistical power calculation for this study, based on our previous data (11), assumes a difference in that study between biomarker positive vs. negative groups of 4 points on the HAMD-21. Given a standard deviation of 6 and the given number of subjects, the current design has a power of ~70% to detect a difference between the treatment groups with a one-sided alpha of 0.05, as appropriate for a proof-of-concept study.

## Results

Twelve subjects were treated with GG and compared to 55 subjects, who were treated with standard of care only (TAU group), in an open label design. After two weeks of treatment the primary outcome variable, the Hamilton-Depression Rating Scale change from baseline showed a significant difference for all groups (*p* < 0.05) and demonstrated a time x treatment interaction (*p* < 0.05), indicating a therapeutic benefit of GG. This was consistent with the item 1 of the HAMD-21 scale-based change. In addition, the CGI-S showed a significant interaction (*p* < 0.05) (Table 2).

**Table 2:** Comparison of changes of laboratory and clinical parameters from baseline to week 2 between treatment groups.

	**GG baseline**			**GG week 2**			**TAU baseline**			**TAU week 2**			**Δ GG**	**Δ TAU**	**Interaction**
	***N***	**Mean**	**SD**	***N***	**Mean**	**SD**	***N***	**Mean**	**SD**	***N***	**Mean**	**SD**			
Age, years	12	37.3	10.9	12	37.3	10.9	55	44.6	15.7	55	44.6	15.7			
BMI, kg/m^2^	12	29.9	7.7	12	30.0	7.7	51	27.8	5.5	51	27.8	5.5			
TST, min	12	409.9	81.5	11	344.2	96.8	53	392.8	72.0	48	391.1	80.1	*p* < 0.08		*p* < 0.05
Heart rate variability	12	70.3	12.0	12	69.1	11.1	51	67.1	14.1	50	64.2	15.3		*p* < 0.05	
Systolic BP, mmHg	12	118.1	14.0	12	119.6	14.3	55	127.2	20.3	53	123.8	18.5		*p* < 0.05	
Mg^2+^, mmol/l	12	0.83	0.05	12	0.98	0.45	53	0.85	0.07	52	0.85	0.07			*p* < 0.05
Na^+^/K^+^ ratio	12	37.5	3.2	12	38.2	3.4	53	37.6	3.1	51	37.1	3.3			
Aldosterone, pg/ml	12	14.3	11.7	11	14.8	7.5	51	22.4	21.8	46	16.3	12.7			
Cortisol, ng/ml	12	4.23	2.17	12	5.38	3.09	54	5.9	3.66	52	6.10	3.92			
HAMD-21	12	26.5	5.4	12	20.2	8.3	55	23.2	7.0	55	18.9	8.3	*p* < 0.01	*p* < 0.01	*p* < 0.03
HAMD (item 1)	12	3.5	0.8	12	2.5	1.6	55	2.9	1.0	55	2.4	1.2	*p* < 0.05	*p* < 0.05	*p* < 0.03
CGI severity	12	4.75	0.75	12	3.83	1.27	54	4.83	0.97	53	4.32	1.22	*p* < 0.02	*p* < 0.01	*p* < 0.05

*Δ GG and Δ TAU, significant difference from baseline (t-test); Interaction, significant interaction of treatment, corrected for gender and rater*.

*TST, total sleep time; BMI, body mass index, BP, blood pressure; HAMD, Hamilton Depression Rating Scale; CGI, clinical global impression*.

In the GG group, blood pressure increased slightly, but not significantly, whereas it dropped in the TAU (RAAS sample) group (*p* < 0.05). Furthermore, HRV declined in the TAU group (*p* < 0.05), but not in the GG group. Heart rate increased in the GG group, but not the standard group. Total sleep time (TST) showed a significant interaction between the groups with a trend to a reduction in the GG group (*p* < 0.08). [Mg^2+^] showed a significant interaction between the groups, most likely as a consequence of Mg^2+^ administration, in the active group. We did not find the expected change in salivary aldosterone or cortisol concentration (Table 2).

**Figure 1:**
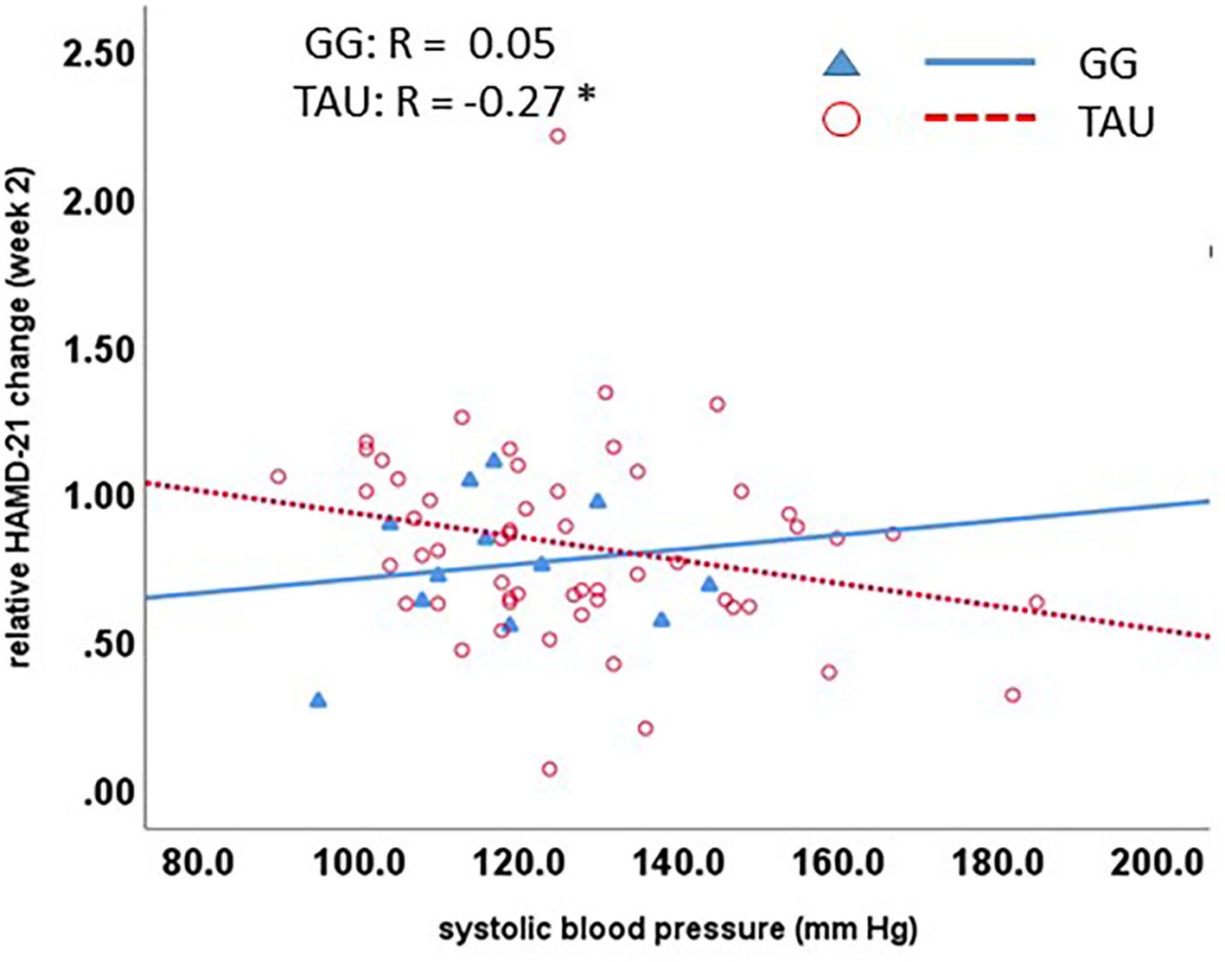
Qualitative patterns for the biomarker-dependent differentiation of clinical outcome between GG-treated and not GG-treated (TAU) subjects. The treatment effect is determined by the ratio of the HAMD-21 at week 2 to that at baseline. A value of 1 refers to no change in HAMD-21; higher values to worsening; and lower values to improvement. Lower systolic blood pressure is related to lesser improvement in the TAU group, but larger improvement in the GG, which leads to a larger differentiation between the groups with lower systolic blood pressure. GG, Glycyrrhiza glabra add-on; TAU, treatment as usual.

**Figure 2:**
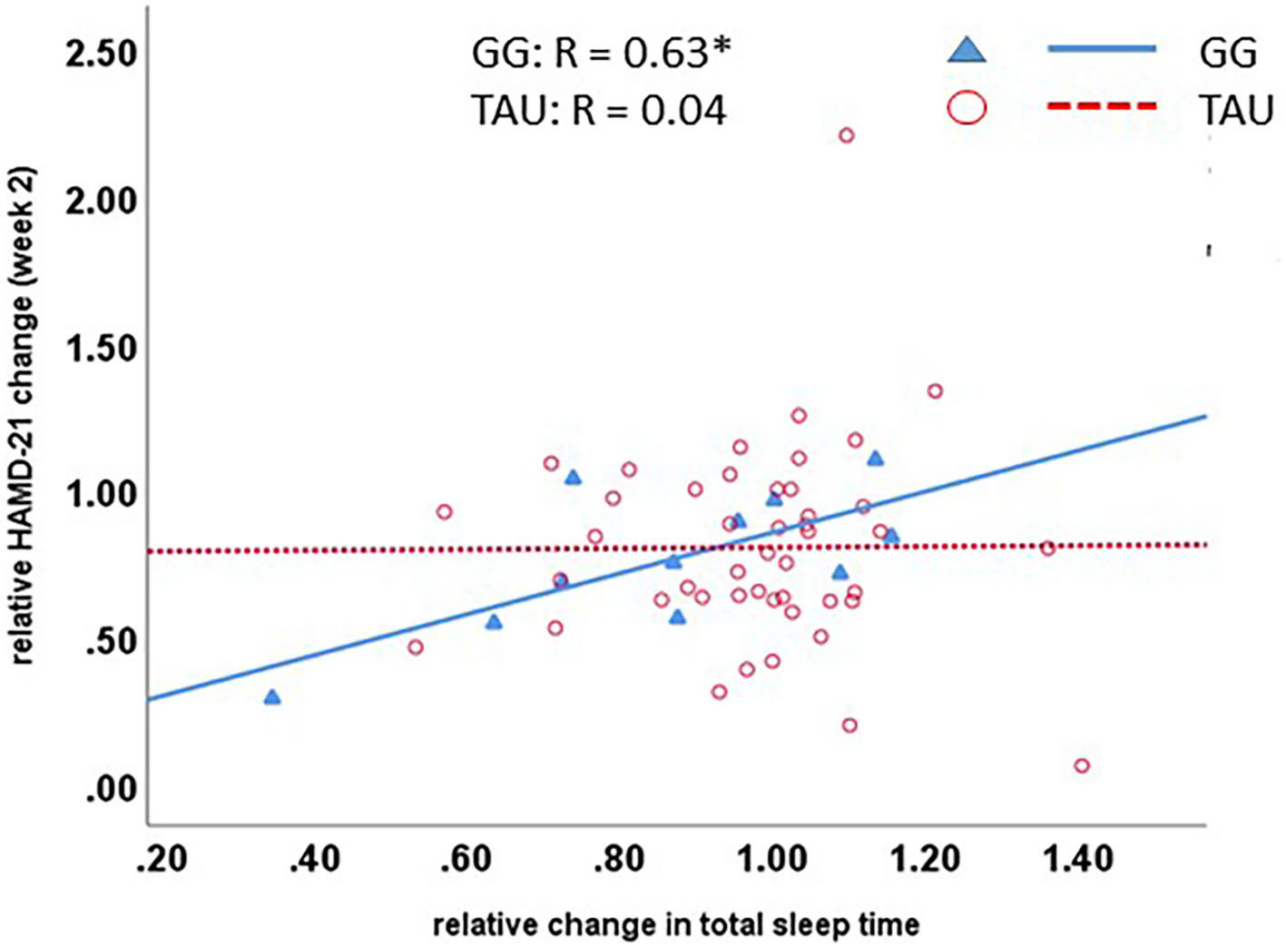
GG-induced biomarker changes are correlated with clinical change. A clinically better outcome, as determined by the HAMD-21 ratio, is significantly correlated with a reduced total sleep time in the GG group. GG, Glycyrrhiza glabra add-on; TAU, treatment as usual.

We calculated correlations in a descriptive way to (1) compare the association of baseline parameters with outcome (Figure 1) and (2) explore changes in biomarkers with that of the HAMD-21 from baseline (R-HAMD-21), all expressed as relative values compared to baseline, as markers of functional target engagement. In the TAU group, but not the GG group, baseline systolic blood pressure was inversely correlated with the relative outcome, meaning that higher blood pressure leads to a more favorable outcome. The correlation lines cross at ~140 mm Hg, which indicates that GG shows descriptively a favorable clinical improvement in comparison to standard treatment below this value (Figure 1).

Furthermore, the relative change in total sleep time was significantly correlated with the clinical change in the GG group, but not the TAU group, in a way that reduction in total sleep time points to clinical improvement (Figure 2).

## Discussion

Alterations in autonomic and neuroendocrine control affect treatment response in patients with depression. Glycyrrhizin is a compound, which was selected to reverse this biomarker pattern and the underlying biology. We could confirm the previously described clinical efficacy of glycyrrhizin in an open label comparison. We could also generate more evidence for the differential relationship between clinical response and systolic blood pressure, which was one prespecified predictor of outcome, but not with the salivary aldosterone/cortisol ratio. We also describe an association of the overall clinical benefit in the GG group with a reduction of total sleep time. This may point to a specific clinically defined sensitive subgroup, i.e., patients with hypersomnia.

In detail, we have described previously that a high ratio of salivary aldosterone/cortisol and low blood pressure may indicate a risk for antidepressant treatment resistance, potentially based on a low peripheral MR activity (11). In an independent population, we found evidence that a low ratio of plasma Na^+^/K^+^ is linked to worse treatment outcome (36). Some of these markers appear to be trait or resilience markers, as they are stable over the treatment interval (12). A trait like reduced sensitivity of the MR has been demonstrated with an endocrine challenge test (17, 18). It was hypothesized that by targeting a mechanism, related to this biomarker pattern, i.e., peripheral MR activation, as regulated partially by 11betaHSD2, may reverse treatment refractoriness. A reduction of plasma aldosterone via inhibition of 11betaHSD2 activity (see Introduction) is regarded as the mediator of this action. As already mentioned in the introduction, the antagonist of 11betaHSD2 and of TLR4, glycyrrhizin, was therefore hypothesized to overcome treatment refractoriness. Indeed, the results of a recent double-blind placebo-controlled study are consistent with our hypothesis, as it demonstrated a superior outcome of glycyrrhizin adjunct to citalopram in comparison to citalopram alone (19). A preferential benefit of patients with higher levels of inflammation, as expressed by C-reactive protein levels > 3 mg/l, was observed in that study. Interestingly, the anti-inflammatory effect of glycyrrhizin and its active metabolite glycyrrhetinic acid is mediated via antagonism at toll-like receptor 4 (TLR4) (29), i.e., the receptor for endotoxin (lipopolysaccharide, LPS). LPS acts synergistically with aldosterone (16) to induce an inflammatory response and depression-like behavior in an animal model. This points to a pro-inflammatory action of aldosterone and, conversely, an anti-inflammatory effect of aldosterone reduction. However, the exact mechanism of glycyrrhizin's anti-inflammatory action and the involvement of aldosterone *in vivo* needs further clarification.

Changes in aldosterone or cortisol were unexpectedly not directly observed in this study. As the salivary collection was performed 12–13 h after the last administration of study medication, the direct effect on the concentration of aldosterone may have disappeared. The observed mentioned functional consequences, i.e., changes in total sleep time and its correlation with clinical improvement, are in line with a suppressed TLR4 activity in subjects with a beneficial outcome (37). Furthermore, a wake promoting effects of NTS stimulation, i.e., of the primary target for aldosterone, has been observed in animal models (38).

Mechanistically, this pattern of changes induced by GG is in line with an increase in peripheral MR activation, leading to an increased blood pressure (relative to the nontreated subjects), and a reduction in activity of the renin–angiotensin aldosterone system. These findings are consistent with an inhibition of the enzyme 11betaHSD2: 11betaHSD2 inhibition leads to a higher access of cortisol to MR, which leads to a suppression of the release of renin and aldosterone (20, 39) without reducing blood pressure. According to our current working hypothesis, lowering aldosterone levels act to modulate the activity of the NTS, which is a key nucleus in autonomic and affect regulation (40). In addition, an increase in cortisol/cortisone ratio can be expected with an inhibition of 11beta HSD2, which, however, appears to be mainly due to a reduction in cortisone concentration along an unchanged cortisol concentration (41).

Interventions, which reduce the activity of the renin–angiotensin aldosterone system, have been demonstrated to influence major depression. Acute treatment with the ACE-inhibitor captopril appears to improve depression in an open-label trial (42) and of lisinopril in a case series (43). In addition, beneficial effects of the angiotensin receptor blocker candesartan have been reported (44). However, direct MR blockade with spironolactone in a placebo-controlled trial did not induce an effect on clinical outcome as an adjunct treatment in depressed patients (45). For the latter finding, it is important to keep in mind that MR blockade is not the same as aldosterone reduction, which is attempted here. In fact, spironolactone leads to an increase in plasma aldosterone in the study by Otte et al. (45). Interestingly, fludrocortisone, which is a mineralocorticoid used to treat postural tachycardia syndrome, induced a more rapid antidepressive response in comparison to placebo-treated subjects in antidepressant treatment responders (45). This is in line with our hypothesis as fludrocortisone leads to a reduction of peripheral plasma aldosterone concentration and is expected to cross the blood brain barrier to only a negligible extent (46).

Genetic and endocrinological findings support the RAAS as a target for antidepressant interventions. We mentioned in the introduction that the gene for the target enzyme 11betaHSD2 shows variability on both genetic and epigenetic levels. Furthermore, polymorphisms within the aldosterone-regulating system have been demonstrated including the genes for angiotensin converting enzyme (ACE) (47), the angiotensin II receptor (ATII) (47, 48), and MR (49, 50), most of which have been linked to stress response or MDD. The gene for 11betaHSD2 itself received attention in the area of blood pressure control, but not yet in psychiatric disorders. However, a number of endocrinological studies, which looked at the putative activity of the 11betaHSD2 enzyme, have been reported. In hospitalized patients with depression, the cortisone/cortisol ratio in plasma was utilized to determine the activity of this enzyme (51). No difference was observed in the population studied. However, the absence of specificity of this test for the 11betaHSD2 vs. 11betaHSD1 limits the interpretation of mentioned results. In addition, the subjects in the Weber et al. study (51) did show marked hypercortisolism, which is not a general finding in major depression, but only observed in about one third of depressed subjects, and mostly related to melancholic features, i.e., those with more severe sleep disturbances. A further study reported a urinary steroid profile in outpatients with depression in line with a reduced activity of the 11betaHSD1 (52) in depressed patients vs. controls. Given that 11betaHSD1 and 11betaHSD2 catalyze opposite reactions, the mentioned results could be regarded as evidence of an overactivity of the 11betaHSD2. A higher activity of this enzyme blocks cortisol from accessing the MR more completely by its more rapid metabolism into the inactive cortisone, which does not activate the MR. This is expected to lead to lower cortisol and higher aldosterone and cortisone levels.

Regarding the specificity of the observed effects, a large cross-sectional study demonstrated important differences in clinically defined subtypes of depression, i.e., atypical depression vs. melancholic depression (53). Atypical MDD is associated with increased inflammation, as expressed by a higher CRP level and an increased activation of the RAAS, as expressed by an increase in the plasma concentration of angiotensin-converting enzyme (ACE). This form also shows higher rates of the metabolic syndrome and a higher BMI (54) and is defined by hyperphagia and hypersomnia. It is interesting to note that reduced sleep duration in our study was associated with the clinical improvement in the GG group, but not the TAU group. This may point to an improvement of a specific symptom of atypical depression and therefore an overlap between the atypical depression subtype and the one, which we characterized on the basis of the here described biomarker pattern.

Important limitations of the study have to be mentioned: we describe the effects on patients treated with GG in an open-label design and compared it to a group of subjects from a different study, but with a very similar design. No randomization into the treatment arms occurred. Overall, the sample of the GG groups is considerably smaller than that of the TAU group. This makes the GG group vulnerable to variability. The comparison of demographic and medication intake data, however, does not indicate a bias toward less therapy resistance in the GG group. Nevertheless, the potential imbalance of the effect of concomitant treatment cannot completely be ruled out. One difference regarding administered substances apart from GG concerns magnesium citrate, which was administered due to the fact that MR activation leads to an increase in excretion of magnesium. We wanted to avoid this, as it had been demonstrated that depletion of magnesium may lead to anxiety and depression-like behavior (55). Clear evidence for the role of magnesium in placebo-controlled trials, however, is missing. Nevertheless, we cannot rule out that the administration of magnesium contributes to the clinical effect. Generally, we would recommend a combination of glycyrrhizin and magnesium compounds. Furthermore, the small sample of the GG-treated subjects made it not meaningful to analyze the influence of gender, which previously had been reported regarding the influence of aldosterone on depression (56). Regarding predictive markers, we could confirm the relationship of blood pressure to clinical outcome in the TAU group, but not its relationship of the saliva aldosterone/cortisol ratio. This needs further clarification. Due to the limited sample size, additional factors, including gender and menopausal status, could not be addressed, which may be of relevance, as atypical depression has a high prevalence in young women.

In conclusion, our current data support the recent hypothesis that the activity of the RAAS and related autonomic characteristics may serve to define a form of depression, which can be targeted with an inhibitor of 11betaHSD2. Our preliminary findings demonstrate that GG expands the effectiveness of antidepressant drugs to a group, which appears to be less sensitive to TAU. However, this hypothesis needs to be confirmed in further randomized double-blind placebo-controlled clinical trials. As the primary action of the GG constituent glycyrrhizin is to inhibit 11betaHSD2 and (directly or indirectly) TLR4, this principal may emerge as a new target for a definable subgroup of patients with depression, if confirmed in future research. A double-blind placebo-controlled study is the next step for further elucidating this new mechanism of action.

## Data Availability Statement

The raw data supporting the conclusions of this article will be made available by the authors, without undue reservation.

## Ethics Statement

The studies involving human participants were reviewed and approved by Ethics committee of the Philipps University Marburg/Germany. The patients/participants provided their written informed consent to participate in this study.

## Author Contributions

HM: conceptualization, formal analysis, writing original draft, writing review and editing, funding acquisition. LL: conceptualization, investigation, data curation, formal analysis, writing review and editing. JH: investigation, data curation, writing review and editing. MB: investigation, data curation, validation, writing review and editing. DJ: investigation, writing review and editing, funding acquisition. MZ: investigation, writing review and editing, supervision. All authors contributed to the article and approved the submitted version.

## Conflict of Interest

HM worked in the past for several pharmaceutical companies, including Acorda Therapeutics and Axovant. He is currently the owner of the consulting company Murck-Neuroscience LLC and holds a patent for the use of glycyrrhizin in the area of depression treatment. The remaining authors declare that the research was conducted in the absence of any commercial or financial relationships that could be construed as a potential conflict of interest.
